# Skin-specific transgenic overexpression of ovine β-catenin in mice

**DOI:** 10.3389/fgene.2022.1059913

**Published:** 2023-01-04

**Authors:** Jiankui Wang, Kai Cui, Guoying Hua, Deping Han, Zu Yang, Tun Li, Xue Yang, Yuanyuan Zhang, Ganxian Cai, Xiaotian Deng, Xuemei Deng

**Affiliations:** ^1^ Key laboratory of Animal Genetics, Breeding and Reproduction of the Ministry of Agriculture, Beijing Key Laboratory of Animal Genetic Improvement, China Agricultural University, Beijing, China; ^2^ Key Laboratory of Feed Biotechnology, Ministry of Agriculture/Feed Research Institute, Chinese Academy of Agricultural Sciences, Beijing, China

**Keywords:** β-catenin, ovine, transgenic mice, overexpression, hair follicles

## Abstract

β-catenin is a conserved molecule that plays an important role in hair follicle development. In this study, we generated skin-specific overexpression of ovine β-catenin in transgenic mice by pronuclear microinjection. Results of polymerase chain reaction (PCR) testing and Southern blot showed that the ovine β-catenin gene was successfully transferred into mice, and the exogenous β-catenin gene was passed down from the first to sixth generations. Furthermore, real-time fluorescent quantitative PCR (qRT-PCR) and western blot analysis showed that β-catenin mRNA was specifically expressed in the skin of transgenic mice. The analysis of F6 phenotypes showed that overexpression of β-catenin could increase hair follicle density by prematurely promoting the catagen-to-anagen transition. The results showed that ovine β-catenin could also promote hair follicle development in mice. We, therefore, demonstrate domestication traits in animals.

## Introduction

The skin is the largest organ in the body and provides a barrier to harmful substances (Proksch et al., 2008). Mammalian skin is composed of multiple layers of epithelial cells covered by a large number of hair follicles and glands (Watt, 2001). The dermal papilla is a relatively large structure in the basal part of a hair follicle (Watt, 2001). Once the dermal papilla is formed, the hair follicle structure becomes very stable throughout its life. This stable state, however, is based on a regular hair follicle cycle of growth (anagen), followed by retrogression (catagen) and then quiescence (telogen) (Li and Tumbar, 2021). Hair follicle formation during the embryonic stage depends on a large number of molecular signals. The interaction of these signaling molecules between the dermis and epithelial cells creates a complex regulatory network during hair follicle development (Hardy, 1992). In postnatal animals, the beginning of the hair follicle cycle is often accompanied by the initiation of a molecular signal in the hair papilla, which causes the transient proliferation of bulge stem cells, further moving them to the root of the hair follicle (Oshima et al., 2001). Previous studies have identified many key molecules that regulate hair follicle development, such as fibroblast growth factors (FGFs), bone morphogenetic proteins (BMPs), and transforming growth factor β 2 (*TGF-β2*) (Millar, 2002). In addition, the Wnt signaling pathway is key in regulating hair follicle morphogenesis. *β-catenin,* the core molecule of the Wnt signaling pathway, plays an important role in hair follicle embryogenesis and *postpartum* development (Huelsken et al., 2001).

*β-Catenin* has two main functions. It activates the expression of target genes downstream of the Wnt pathway by activating the pathway and also mediates cell-cell adhesion (Schuman and Murase, 2003). *β-catenin* plays a diverse role in the Wnt signaling pathway and interacts with many proteins, such as *APC*, *AXIN*, and *GSK-3β* (Ozawa et al., 1989). In the classical Wnt signaling pathway, *β-catenin* is transported to the nucleus and binds to the *TCF/LEF* family, increasing the transcriptional activity (Doumpas et al., 2019). Previous studies have shown that *β-catenin* is essential for hair follicle development (Narhi et al., 2008). *β-catenin* mRNA expression increased during hair follicle morphogenesis at the embryonic stage (Tsai et al., 2014). In contrast, blocking *β-catenin* expression failed to form normal hair follicles (Liu et al., 2014). *In vitro* studies have indicated that knockout of *β-catenin* produces two phenotypes: a lack of substrate during hair follicle embryogenesis and hair loss during the first hair follicle cycle (Huelsken et al., 2001). Compared to that in wild-type mice, overexpression of incomplete *β-catenin* in mouse skin tissue could lead to an increase in the number and size of hair follicles and promote hair follicles to enter the anagen phase from the quiescent phase (Doumpaset al., 2019b). The “bulge-activation hypothesis” considers that when a signal is transferred from the dermal papilla and activates the stem cells in the bulge of the hair follicle, proliferation of the hair follicle stem cells is stimulated, and the follicles enter a new growth period. *β-catenin* plays a crucial role in this process (Li and Tumbar, 2021).

Aohan fine wool sheep, bred for their wool and meat, are well known for their slaughter rate and wool fineness (Liu et al., 2014). In recent years, the demand for superfine wool in the wool textile industry has increased rapidly. Improving wool density and fineness is a top priority in the production of high-quality wool (Han et al., 2021). In this study, we explored whether ovine *β-catenin* can be used as an exogenous gene to affect the development of hair follicles in mice, and the results from the transgenic mice model gave us a chance to understand the molecular mechanisms of skin-specific transgenic overexpression of ovine *β-catenin* in sheep (Wang et al., 2019). This will aid further research into the critical role of *β-catenin* in improving the wool quality in fine-wool sheep breeds.

## Materials and methods

### K14-β-catenin (ovine)-IRES-EGFP plasmid construction

The *β-catenin* was amplified by PCR from the cDNA of sheep skin, which contains SacII and BamHI sites, with the primer shown in Table 1. The purified products were inserted into PMD19T plasmid vector (TAKARA, Japan) and sequenced at SinoGenoMas Co., Ltd. (Beijing, China). The 2.0 kb K14 promoter with AseI and AfeI sites obtained by PCR was cloned into PMD-19T vector and sequenced as above. In order to remove the CMV promoter of pIRES-EGFP, the restriction enzyme AfeⅠ and AseⅠ were used to digest and the human K14 promoter was inserted into the AseⅠand AseⅠ site to produce the skin-specific expression plasmid K14-IRES-EGFP. Afterwards, the ovine *β-catenin* gene was inserted into K14-IRES-EGFP plasmid by digesting with restriction enzyme BamHⅠ and SacⅡ. The resultant plasmid K14-*β-catenin*-IRES-EGFP was then sequenced (SinoGenoMax Co., Ltd., Beijing, China).

**TABLE 1:** Primer sequences used to produce transgenic mice. F1, R1 for the amplification of β-catenin. F2, R2 for the amplification of promoter K14. F3, R3 for the qRT-PCR of transgenic mice. F4, R4 for the amplification of housekeeping gene. F5, R5 for the identification of positive mice. F6, R6 for the synthesis of probe of Southern blot. The underlined bases are the restrict enzyme sites.

Primers	Sequence (5′-3′)	Fragment length	Ta value(C)	
*β-catenin*	F1	TCC​CCG​CGG​CGG​AGA​CGG​AGC​AAG​GT	2656bp	56
	R1	CGC​GGA​TCC​GCA​AGC​AAA​GTC​AGT​ACC​AT		
*K14*	F2	CAT​TAA​TAT​CCC​TGC​AGA​AGA​AGG​AGA​C	2000bp	55
	R2	ATA​AGC​GCT​GGC​TGA​GTG​AAG​AGA​AGG		
qRT-PCR	F3	AGC​GTC​GTA​CAT​CTA​TGG​G	368bp	59
	R3	ATA​ATC​CTG​TGG​CTT​GAC​C		
*GAPDH*	F4	GTC​CGT​TGT​GGA​TCT​GAC​CT	245bp	59
	R4	TGC​TGT​AGC​CGA​ATT​CAT​TG		
Identification	F5	TCACCGTAGGGAGGAAAT	1226bp	56
	R5	TAGCGTCTCAGGGAACAT		
Probe synthesis	F6	CAAGAAAGCCCAAAACAC	739bp	57
	R6	TAGCGTCTCAGGGAACAT		

### Generation of transgenic mice

In order to generate the linear DNA fragments, the AseⅠ was used to digest the plasmid K14-*β-catenin*-IRES-EGFP, then the restricted DNA products were resuspended in sterile ddH_2_O at a concentration of 20 ng/μL. Then, the products were injected into the male pronuclei of zygote of C57BL/6 by Shanghai Research Center for Model Organisms. The F1 generation mouse were produced by mating positive founder mouse (carry K14-β-catenin-IRES-EGFP fragments) with wild type.

### Polymerase chain reaction and southern blot analysis of F0 and F1 mice

Genomic DNA was extracted from tail of the transgenic/control mouse by using the TIANamp Genomic DNA Kit (TIANGEN, Beijing, China). PCR assay was performed by using the primers shown in Table 1. The 1226 bp PCR product contains partial K14 promoter and partial ovine *β-catenin* DNA sequence. The PCR procedure: 5 min at 95°C, 35 cycles of 30 s for 95°C, 30 s for 58°C and 72°C for 1 min, 72°C for 10 min and hold at 4°C forever. The further southern blot assay, digestion: the BstEⅡ was selected for digested the DNA (60°C, 20 h), which is obtained from mouse tail. The probe (739 bp) amplified by primer (F6 and R6 in Table 1) across promoter and β-catenin sequence, was labeled with the PCR digoxigenin probe synthesis kit (Roche). The DIG-High Prime DNA Labeling and Detection Starter KitⅡ was used for washing and hybridization (Roche, Basel, Switzerland).

### Analysis of transgene expression of F2 and F3 mice

Total RNA of different tissue (heart, liver, lung, kidney, skin, muscle and adipose) were isolated by using an RNAprep Pure Tissue Kit (TIANGEN, Beijing, China). According to the guidelines in the kit, 1–2 μg total RNA was used to synthesized the first-stand cDNA using ImPromⅡ Reverse Transcription System (Promega, Beijing, China). qRT-PCR was performed (primers in Table 1) and the analysis of *β-catenin* expression was evaluated by quantitative real-time PCR using real-time PCR detection system (Bio-Rad, United States). The *β-actin* was used as internal references. The results of *β-catenin* expression related *β-actin* were test in triplicate. The final results were generated using the 2^−△△CT^ method (Livak and Schmittgen, 2001).

### Protein analysis of F3 mice

Samples from the tail of transgenic mice were used for fluorescence microscope analysis of green fluorescent protein (GFP). For western blot analysis, according to the kit guidelines of RIPA buffer (BiYoTimel, Suzhou, China), protein was extracted from different tissues of transgenic and control mouse. The concentration of extracted protein was measured by BCA Protein Assay Kit (BiYoTime, China) and the bovine serum albumin was used as a standard. Each lane contained an equal amount of protein (20 μg per lane) and was subjected to sodium dodecyl sulfate-polyacrylamide gel electrophoresis. Then transferred onto the polyvinyl-difluoride membranes at 120 V for 90min. The membranes were incubated with 1:1000 primary anti-β-catenin (abcam, E247, United States), and β-actin (Beyotime, AF5001, China). After 1 hour blocking, wash five times with Tris-buffered saline, each time last 5 min. Horseradish peroxidase-conjugated secondary antibody (1; 10,000) was used to incubated the membranes (BiYoTime, Suzhou, China). Then, the membranes were visualized by using enhanced chemiluminescence system after wased six times with TBST (each time last 5 min).

### Histology analysis of F6 mice

The 8 micron-thick of skin sections were obtained by freezing microtome (LEICA, CM 1900, Germany). The hematoxylin and eosin staining was performed using a Hematoxylin and Eosin Staining Kit (Beyotime, China, C0105S). For analysis of the hair follicle density, the Zeiss dissecting microscope and Zesiss light microscope (Carl; Zeiss, Germany) were used, the hair follicle numbers of per one square millimeter of mice back skin section was counted and the average of three areas of each samples were identified as the hair follicle density.

### Statistical analysis

Differences between transgenic and non-transgenic mice groups were tested using independence Student’s t-test. In this study, two-sided *p* values were identified significant while lower than 0.05.

## Results

### Identification of F0 and F1 transgenic mice

**FIGURE 1:**
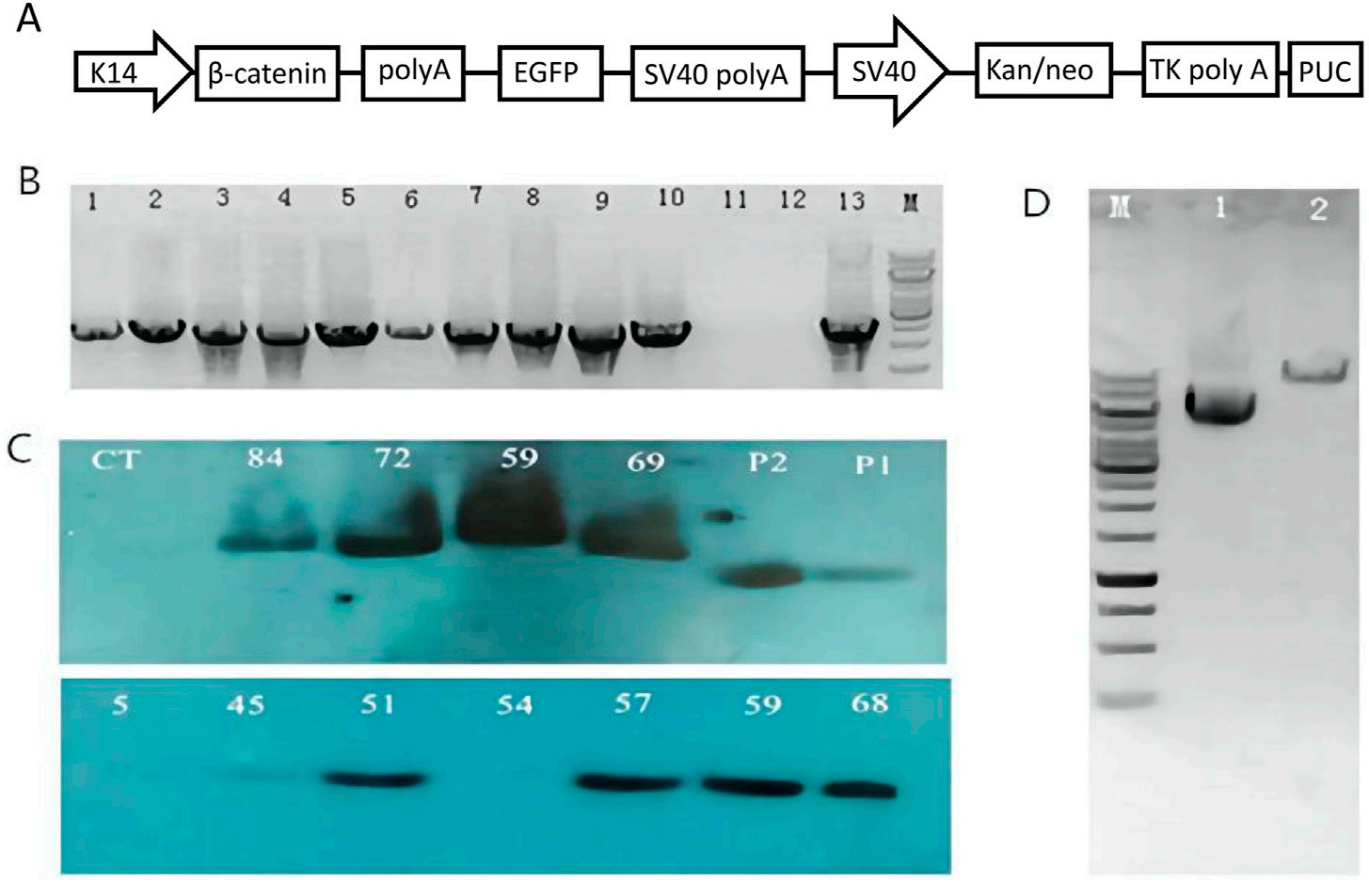
Generation of ovine β-catenin transgenic mice (F0). **(A)** Schematic diagram of transgenic vector (pK14-β-catenin-EGFP). **(B)** Agarose gel analysis on PCR products from genomic DNA extracted by transgenic founder mice tail. The PCR products from mouse number 5, 45, 51, 54, 57, 59, 68, 69, 72, and 84 are shown in well #1 to #10 respectively; the PCR product with H_2_O as a control temple is shown in well #11; the negative PCR product with DNA extracted from wild type mouse tail is in well #12; PCR product using transgenic vector DNA as template is shown in well#13. **(C)** Southern blot with DNA from transgenic mice skin. WT, a sex- and age-matched wild-type control; p1, p2 plasmid controls. **(D)** Analysis of β-catenin Vector DNA by AseI digestion on 1% agarose gel. Lane 1: Vector DNA without any restriction enzyme digestion; Lane 2: Vector DNA digested by AseI; M: 1 Kb DNA ladder.

In this study, a pK14-β-catenin-IRES-GFP vector was successfully constructed (Figure 1A). The vector was linearized by Ase I digestion and then microinjected into the nucleus of mouse zygotes (Figure 1D). 480 fertilized oocytes were injected with linearized vector fragments, and the injected oocytes were then transplanted into 24 recipient mice. A total of 93 founder mice were obtained, of which 10 were identified as transgenic mice by PCR (Figure 1B). The presence of the exogenous ovine *β-catenin* gene was further confirmed by Southern blot (Figure 1C), and eight of the ten PCR-positive mice were confirmed to be transgenic mice carrying the ovine *β-catenin* gene. Eight transgenic mice were healthy and defect-free. Eight transgenic lines were established by crossing the founders with wild-type C57BL/6 mice. It was then expanded to the sixth generation. In this process, PCR and Southern blot techniques were used to identify transgenic mice.

### Characterization of ovine *β-catenin* expression in transgenic mice

**FIGURE 2:**
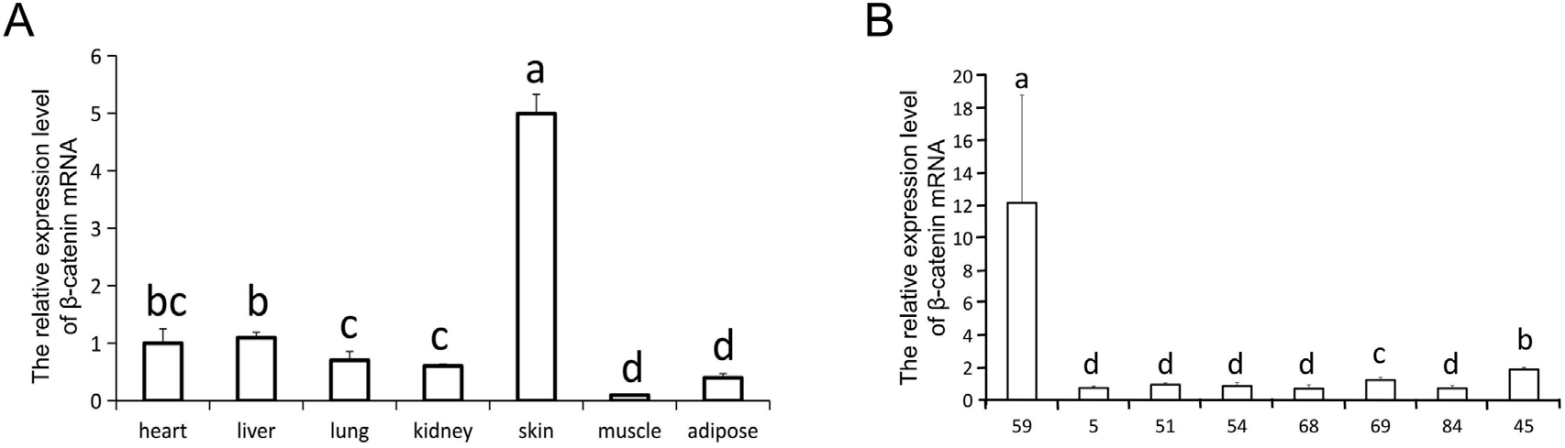
The relative expression level of β-catenin mRNA. **(A)** The expression of β-catenin mRNA in skin of different transgenic mouse lines (F0). 45 is a wilde-type control line. **(B)** qRT-PCR analysis of the β-catenin mRNA expression in different tissues of line 59 (F0). The relative quantity of β-catenin mRNA in **(A)** and **(B)** were determined *via* qRT-PCR using 2^−ΔΔCT^ method, with GAPDH as the internal control. Each experimental group contained three replicates and qRT-PCR was performed in triplicate for each sample. Bars with common lowercase letters had no significant difference at the 5% level.

To assess the characteristics of ovine *β-catenin* expression in transgenic mice, we used qRT-PCR to detect *β-catenin* mRNA levels in different tissues of transgenic mice. The results showed that the expression level of *β-catenin* mRNA in the skin of transgenic mice of line 59 was significantly higher than that in the skin of transgenic mice of other lines (*p* < 0.01) (Figure 2A). We investigated the tissue specificity of ovine *β-catenin* transgene expression in F3 transgenic mice of line 59, and the results showed the transgene expression level in the skin was significantly higher than in other tissues (*p* < 0.01) (Figure 2B). The results indicated that the transgenic mouse of F1 could pass the foreign ovine *β-catenin* onto their offspring, and ovine *β-catenin* was specifically expressed in the skin of transgenic mice.

### Tissue-specific expression of the *β-catenin* protein in transgenic mice

**FIGURE 3:**
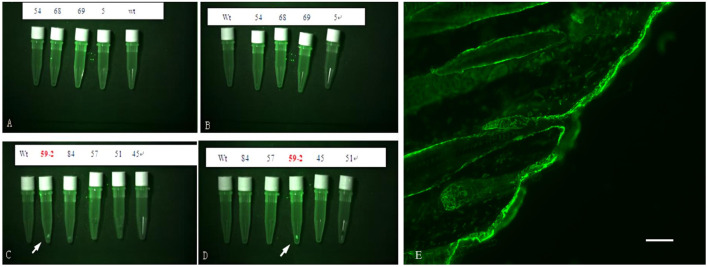
The detection of Green Fluorescent Protein (GFP) in skin of F0 transgenic mice. **(A)**, **(B)**, **(C)** and **(D)** are the fluorescence microscope analysis of the skin of the transgenic mice. Wt is wilde-type control. GFP was detected in line 59, indicated with white arrow. **(E)** The fluorescence Protein in skin tissue of ovine β-catenin transgenic mice, the scale bar: 60 μm.

**FIGURE 4:**
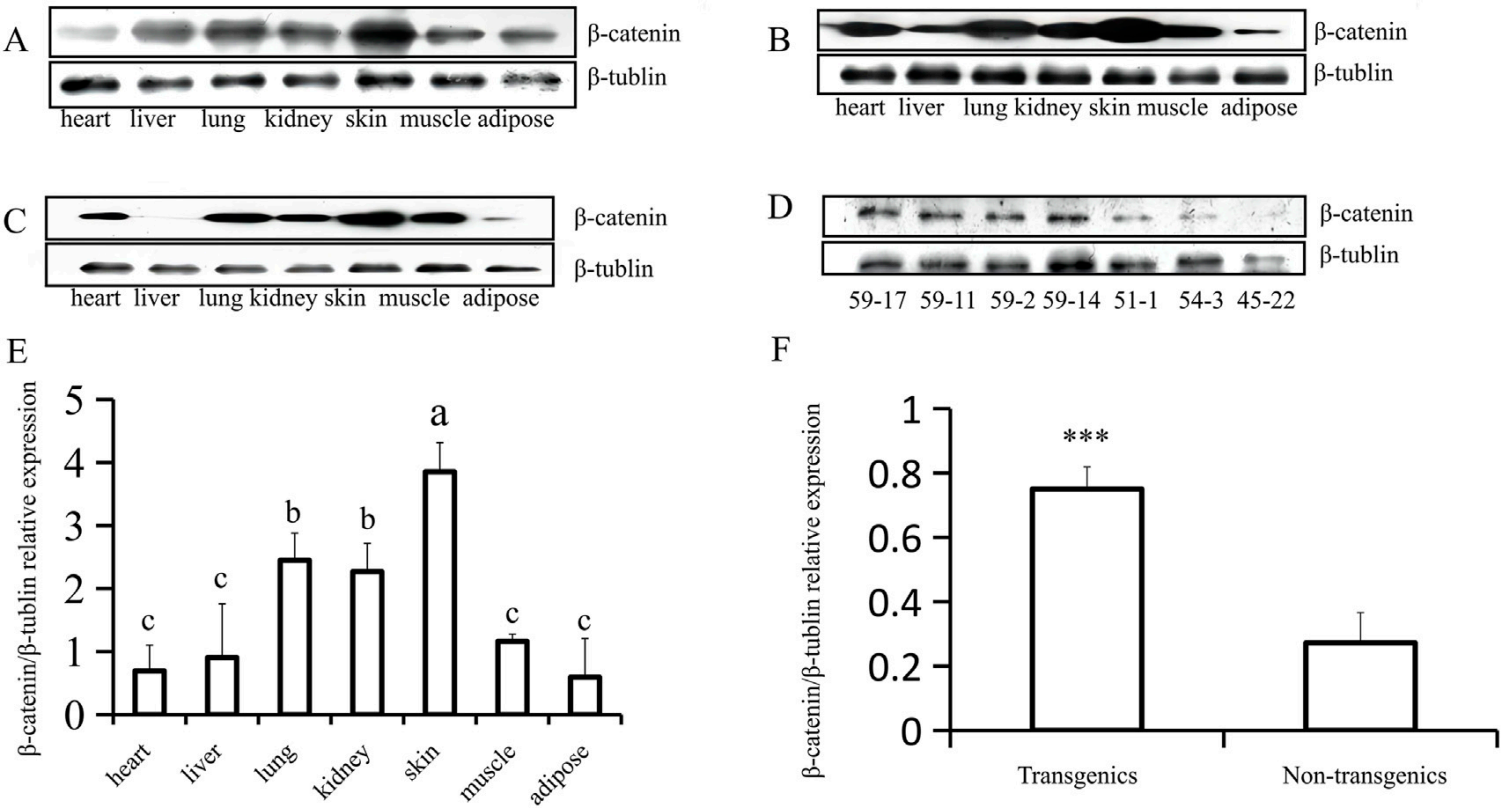
Western-blot analysis of β-catenin protein expression in heart, liver, lung, kidney, skin, muscle and adipose (F1). **(A) (B)** and **(C)** The analysis of β-catenin protein expression in different tissues of three transgenic mice (59–17, 59–2, and 59–11). **(D)** The expression of β-catenin protein in transgenic (59–17, 59–11, and 59–2) and non-transgenic mice (51–1, 54–3, and 45–22). **(E)** The statistics of β-catenin protein expression in different tissues of transgenic mouse lines, the bars with common lowercase letters had no significant difference at the 5% level. **(F)** Comparative analysis of β-catenin protein expression between transgenic and non-transgenic mice. ***, *p* < 0.001.

Analysis using fluorescence microscopy showed that green fluorescent protein (GFP) was expressed in the skin tissue in mice from line 59 but not in the other lines (Figures 3A–E). The western blot results show that the *β-catenin* protein could be detected in the heart, liver, lung, kidney, skin, muscle, and adipose tissues (Figures 4A–C). The highest expression level of the *β-catenin* protein was observed in the skin compared with that in other tissues (*p* < 0.01). These results indicated that the *β-catenin* protein expression level in the skin of transgenic mice of line 59 was significantly higher than that in the other lines (Figures 4D,F) (*p* < 0.01). In addition, the *β-catenin* protein was expressed at significantly higher levels in the skin of transgenic mice than in that of non-transgenic mice (Figures 4A–C,E).

### The significant dosage effect of *β-catenin* copy number and hair follicle traits

**FIGURE 5:**
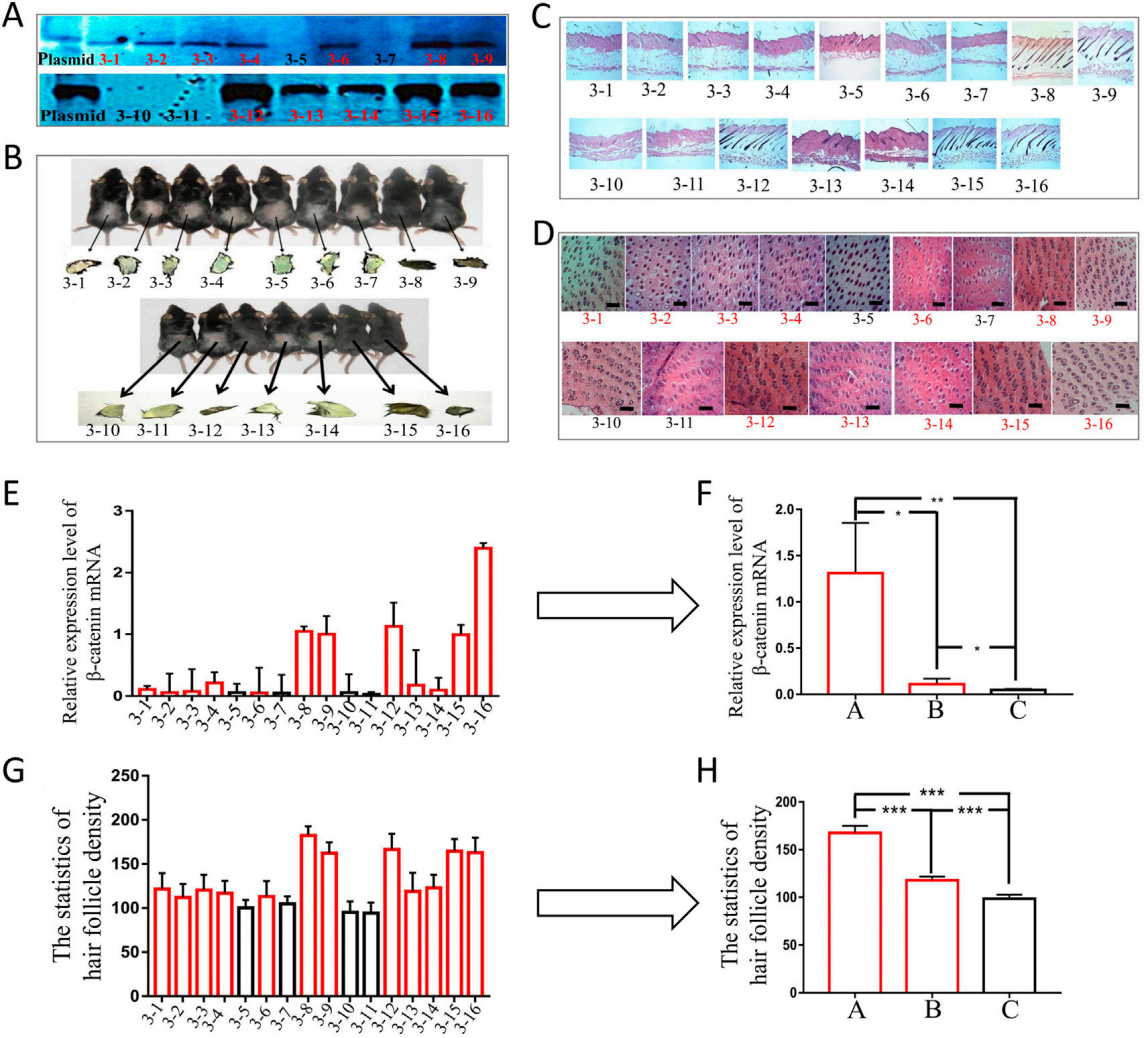
The phenotypic analysis of F6 transgenic mice by overexpressing ovine β-catenin. **(A)** Southern-blot of two transgenic families, the thicker black bands represent higher copy number of foreign gene (ovine β-catenin) while the thinner bands represent lower copy number of ovine β-catenin. **(B)** The two transgenic families of mice and their skin color. **(C)** The longitudinal slices of transgenic mice skin of the two families. **(D)** The transection sections of transgenic mice skin of the two families, the scale bar: 600 μm. **(E)** The expression level of β-catenin mRNA in transgenic mice of the two families. **(F)** The analysis of β-catenin mRNA levels of transgenic mice that were divided into three groups (A: high copy number group, **(B)** low copy number group and **(C)** non-transgenic siblings). *, *p* < 0.05; **, *p* < 0.01. **(G)** Statistical analysis of hair follicle density in transgenic mice of the two families. **(H)** The analysis of hair follicle density of transgenic mice that were divided into three groups (A: high copy number group, **(B)** low copy number group and **(C)** non-transgenic siblings). ***, *p* < 0.001.

To carry out phenotypic analysis, we selected blot transgenic and non-transgenic mice of the same age and sex from three families of F6. Southern was used to not only identify the transgenic mice but also determine the different copy numbers among transgenic mice by the depth of band gray levels (Figure 5A). The results indicated that the greater the copy number of the foreign gene (ovine *β-catenin*) between transgenic and non-transgenic mice, the more significant the differences in *β-catenin* expression (Figures 5E,F) and hair follicle density (Figures 5D,G,H). In addition, transgenic mice with a higher copy number of ovine *β-catenin* showed an earlier anagen phase on postnatal day 56 (telogen) of the dorsal skin (Figures 5B,C).

## Discussion

In our previous study, we cloned the *β-catenin* cDNA sequence (GenBank accession number KC668410) from Aohan fine wool sheep. Homology analysis showed that the *β-catenin* gene is highly conserved among species, and the amino acid sequence of the *β-catenin* protein is 99% conserved among species (Cui et al., 2014). To further explore whether ovine *β-catenin* can be used as a foreign gene to affect hair follicle development, we generated transgenic mice and expressed the ovine *β-catenin* protein specifically in their skin tissue. The results of the qRT-PCR analysis showed that *β-catenin* mRNA could be detected in various tissues. This was because the primers cannot distinguish the endogenous and exogenous *β-catenin* sequences since sheep and mouse share 89.39% of the CDS sequence of *β-catenin*. The expression of *β-catenin* mRNA in other tissues may be a result of endogenous *β-catenin*. Of the different tissues tested, *β-catenin* mRNA was most prevalent in the skin, indicating that the foreign gene was successfully expressed in the target tissue. The western blot and qRT-PCR results suggested that the *β-catenin* protein can be passed down from F1 to their offspring and that *β-catenin* protein is specifically expressed in the skin of the transgenic mice. Green fluorescent protein detected in the skin of line 59 showed that the internal ribosome entry site (IRES) mediated the exogenous gene and GFP expression together, which could improve the detection accuracy of transgenic-positive animals (Kozak, 2005).

Hair follicles are formed during embryogenesis and show regular hair follicle cycles throughout their lifecycle (anagen, catagen, and telogen) (Hardy, 1992; Paus et al., 1999). As a key molecule in the Wnt signaling pathway, *β-catenin* is highly expressed in hair follicles (Ozawa et al., 1989; Goding, 2000). Transient activation of *β-catenin* signaling in skin keratinocytes can promote hair follicle growth (Van Mater et al., 2003). Frozen sections of skin showed that the overexpression of *β-catenin* in the skin can enhance follicle density, similar to previous research (Gat et al., 1998). Immunohistochemical analysis of transgenic mice showed that *β-catenin* was expressed in the dermal papilla and inner root sheath of the hair follicles (Wang et al., 2012). Previous research has revealed that *β-catenin* is highly conserved during biological evolution (Fuchs et al., 2001). The skin-specific expression of ovine *β-catenin* can increase wool follicle density, leading to higher wool production in transgenic sheep (Wang et al., 2019), which agrees with the results of this study.

In conclusion, we successfully generated transgenic mice with skin-specific overexpression of ovine *β-catenin*. The recombinant plasmid exhibited good genetic stability, and the exogenous gene could be transmitted from F1 to F6. The transgenic mice were healthy and had no defects. We demonstrated that transgenic mice overexpressing ovine *β-catenin* showed a high density of hair follicles and an early-entered anagen (Grigoryan et al., 2008).

## Data Availability

The original contributions presented in the study are included in the article/Supplementary Material, further inquiries can be directed to the corresponding author.
